# The Application of Radiolabeled Mesoporous Silica Nanoparticles in Molecular Imaging

**DOI:** 10.3390/molecules31122181

**Published:** 2026-06-22

**Authors:** Aleksandra Lis, Martyna Orłoś, Paweł Szymański

**Affiliations:** 1 Department of Pharmaceutical Chemistry, Drug Analysis and Radiopharmacy, Faculty of Pharmacy, Medical University of Lodz, Muszynskiego 1, 90-151 Lodz, Poland; aleksandra.lis@umed.lodz.pl (A.L.); martyna.orlos@student.umed.lodz.pl (M.O.); 2 Department of Medical Biology, Military Institute of Hygiene and Epidemiology, Kozielska 4, 01-163 Warsaw, Poland

**Keywords:** drug delivery, mesoporous silica nanoparticles, nanotechnology, radionuclide, theranostics

## Abstract

In medicine, nanoparticles are used for various purposes, including theranostics, imaging, diagnostics, drug delivery, tissue regeneration and targeted cancer treatments, and to minimize the harmful side effects associated with conventional therapies. Target-specific biomolecules, such as silica nanoparticles (SiNPs) labeled with metallic radionuclides, are becoming increasingly popular. The choice of radionuclide is based on its nuclear properties. Silica has several advantages for nanoparticle synthesis, including high biocompatibility, the capacity for drug encapsulation due to its porous structure, and the potential for extensive surface functionalization, including radiolabeling for imaging and therapeutic applications. A radionuclide can be attached to a silica nanoparticle either directly or through the use of chelators or polymers. Additionally, the capability to encapsulate therapeutic agents within such systems offers significant potential for the development of targeted therapies. This study aims to provide a comprehensive overview of recent developments in the radiolabeling of silica-based nanoparticles, with a focus on their application in nuclear medicine, particularly in diagnostic imaging and targeted radionuclide therapy. Theranostics employs a range of imaging modalities to guide and monitor therapeutic interventions. Principal techniques include positron emission tomography (PET), single-photon emission computed tomography (SPECT), magnetic resonance imaging (MRI), and Optical Imaging (such as fluorescence and bioluminescence). These imaging methods enable precise visualization of pathological sites, facilitate tracking of therapeutic agent distribution, and permit real-time assessment of treatment efficacy.

## 1. Introduction

Both nanotechnology and radiopharmacy are multidisciplinary fields that have been developing rapidly over the years. Nanotechnology offers considerable potential in science by exploiting various biological, chemical and physical processes occurring at the nanometer scale; however, such a dramatic size reduction translates into altered chemical and biological properties resulting in new bond types and targeted drug delivery [1]. The discipline concerning the use of nanoparticles or nanomaterials in areas of medicine is nanomedicine [2].

Mesoporous silica nanoparticles can possess good biocompatibility, large surface area, drug loading ability, easy functionalization and low toxicity. These properties are of particular value in inter alia pharmacy, biomedicine, industrial coatings, and novel self-assembling materials [3]. In medicine, nanoparticles are frequently used in imaging, diagnostics, drug delivery, tissue engineering or targeted cancer therapy with the aim of reducing the side effects of toxic therapeutics; they can also be labeled with radioisotopes, which also makes them particularly suitable for use in imaging and theranostics. The chemically reactive surface of SiNPs can be easily functionalized through silanization: this allows the attachment of various functional groups, and the subsequent conjugation of chelators for radiolabeling [4]. In addition, intrinsically radiolabeled SiNPs can also be prepared without the use of chelators; this approach takes advantage of the strong coordination between radiometals and electron-donating atoms (such as oxygen, sulfur, or nitrogen) found on the surface of the SiNPs [5].

The role of a radiopharmaceutical is to deliver a radionuclide to a target site using carriers such as antibodies, peptides, and small particles, including nanoparticles on the nanometer scale. Moreover, the precise deposition of high-energy radiation emitted by radionuclides within targeted cells results in cytotoxic effects through the induction of single- and double-strand DNA damage, ultimately leading to cell death [6]. The ideal theranostic nanoparticle is characterized by safety, lack of immunogenicity, targeted accumulation in pathological tissues, diagnostic imaging capability, effective therapeutic delivery, and efficient clearance or biodegradation after fulfilling its function.

This article provides a detailed analysis of the isotopes employed for the radiolabeling of mesoporous silica nanoparticles (MSNs), detailing their structural characteristics and surface functionalization strategies. It explores their applications in nuclear medicine and Oncology, with particular emphasis on their role as carriers for the targeted delivery of anticancer therapeutics.

## 2. Mesoporous Silica Nanoparticles

Nanoparticles (NPs) are solid-state materials which can be arranged into various groups based on their size, shape, morphology, and physical and chemical properties. Some examples include nanoparticles based on carbon structures, ceramics, polymers, metals, semiconductors, and lipids. Overall, nanoparticles can be broadly classified into two main categories according to their composition: organic and inorganic nanomaterials. NPs are commonly used in different field of biomedicine such as drug delivery, fotothermal therapy, sensing or theranostics [7]. Mesoporous silica nanoparticles (MSNs) have garnered considerable interest owing to their unique physicochemical characteristics, including tunable and well-defined pore size, high pore volume and specific surface area, facile surface functionalization, the presence of both internal and external pore architectures, controllable pore gating mechanisms, favorable biocompatibility and biodegradability, robust mechanical and thermal stability, high loading capacity, and excellent colloidal stability in aqueous media [8].

On the other hand, the safety profile of nanoparticles is further complicated by the possibility that their surface properties may induce inflammatory reactions or undesired immune responses. These factors underline the importance of comprehensive risk assessment and appropriate regulatory supervision in the development and use of nanomaterials [9].

The nanoparticle surface may be easily modified with peptides and other biomolecules, or by polymers such as polyethylene glycol (PEG), a procedure known as PEGylation. These changes reduce the risk of opsonization after intravenous administration by forming a steric barrier [10]. Moreover, the use of ligands enables the active transport of molecules to affected areas and reduces the risk of aggregation and agglomeration. PEGylation is commonly used during structural modifications and influences the circulation time of SiNPs in the human body. Where the surface has been modified, the size of the resulting modified particle also should be taken into account [11]. Fang et al. [12] report a correlation between size and risk of opsonization: approximately 34% of NPs 240 nm in diameter were opsonized compared to 6% of those measuring 80 nm. In 1995, a PEGylated liposomal formulation loaded with Doxorubicin (Doxil/Caelyx) was approved for use as a medicine [13].

It has long been recognized that the hydrolysis and condensation of tetraethyl orthosilicate (TEOS) generate a large number of silanol (–Si–OH) groups on the surface of amorphous silica particles [14]. At pH values higher than the silica isoelectric point (approximately pH 2–3), surface –Si–OH groups are converted into negatively charged –Si–O^−^ groups through deprotonation [15]. Radiolabeling of SiNPs with radiometals proceeds through the formation of coordination complexes resulting from Lewis acid–base interactions. In this process, a radiometal ion (Lewis acid) is coordinated by ligands acting as Lewis bases, which donate electron pairs via coordination bonds. The strength of the interaction between the metal center and ligands largely determines labeling efficiency and in vivo stability. SiNPs can be readily functionalized with various chelators to bind radiometals; alternatively, their oxygen-rich surface makes them intrinsically suitable as hard Lewis bases for coordinating hard radiometal ions [16].

**Figure 1:**
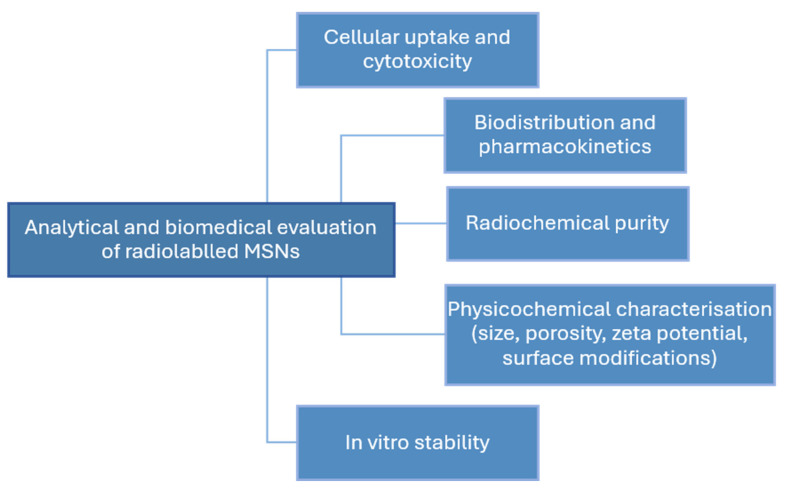
Crucial evaluations to understanding radiolabeled MSNs in biological systems and its implications for safety, efficacy and therapeutic index optimization.

The shape of the nanoparticles is also very important in case of blood circulation. Spherical nanoparticles and those with a short rod shape have a shorter circulation time in the bloodstream, unlike those that are long-rod-shaped [17]. The most important aspects related to the safety of mesoporous silica nanoparticles are summarized in Figure 1.

### Radioisotope-Labeled Mesoporous Silica Nanoparticles

**Figure 2:**
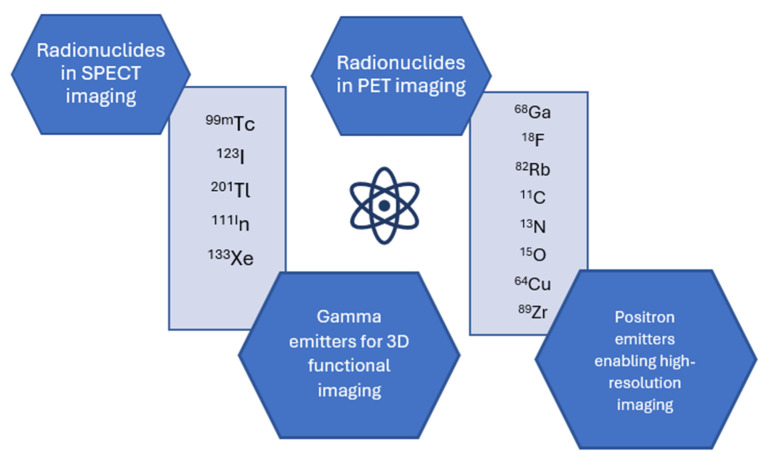
Classification of commonly used radionuclides according to their applications and type of radiation emission in imaging. The atomic symbol is used for illustrative purposes only and does not represent any experimental variable.

Nanomedicine has considerable potential for cancer diagnosis and therapy. Nuclear medicine, a discipline that involves the release of high-energy particles such as protons, neutrons, or α- or β-radiation, or electromagnetic waves such as X-rays and γ-rays, has transformed the diagnosis and treatment of cancer [18]. Tumor cells can be selectively destroyed using α, β-, and Auger electron emitters [19]; auger electrons (AEs) are very low-energy electrons emitted by radionuclides that decay by electron capture (e.g., ^11^In, ^67^Ga, ^99m^Tc, ^195m^Pt, ^125^I and ^123^I) [20]. The choice of radionuclide depends on the desired duration of the imaging agent, as well as the decay type of the radionuclide [21]. Table 1 presents selected isotopes employed in conjunction with mesoporous silica nanoparticles, along with their half-lives, radiation emission characteristics, and applications in imaging. Mesoporous silica nanoparticles can be labeled with various types of radioisotopes (Figure 2). Those with a long half-life are better suited for targeted therapy and complex radiolabeling procedures, while short half-lives are typically used for diagnostic purposes [22].

**Figure 3:**
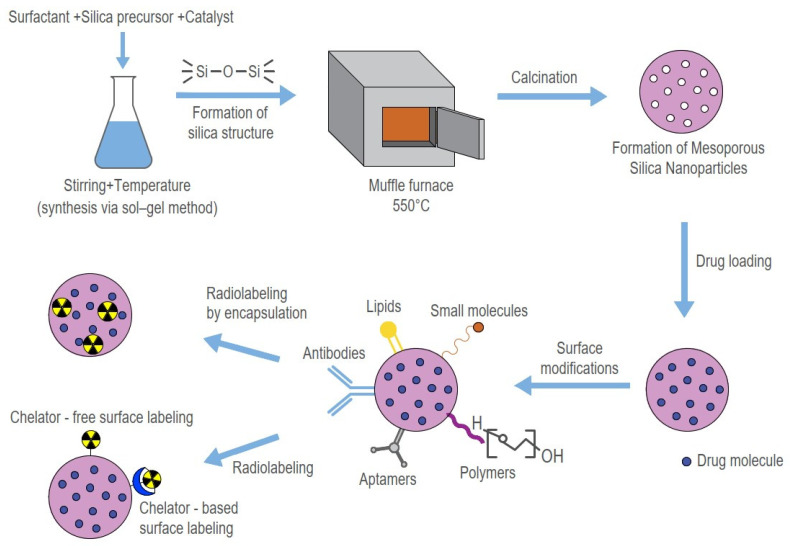
Schematic of MSNs synthesis via sol–gel method, including drug loading, surface modifications and radiolabeling.

Ideally, radiolabeling is performed by making minimal changes in the original properties of the silica nanoparticle; the procedure should be easy, fast and highly efficient. Radioisotopes can be bound to nanoparticles in different ways. One method involves the conjugation of a radionuclide directly to the surface of the particle by a Lewis acid–base reaction. The central radiometal ion (or cation) forms coordination bonds with surrounding neutral molecules or anions, known as ligands, which donate electron pairs to stabilize the metal complex. In this system, the ligands act as Lewis bases, while the radiometals function as Lewis acids; the interaction between the Lewis bases and acids determines the radiolabeling efficiency and stability. Another option is to load the radionuclide into the core of the nanoparticle using a linker, which might be a chelator such as diethylenetriaminepentaacetic acid (DTPA) or a polymer such as PEG [16]. A schematic presentation of different types of radiolabeling is depicted in Figure 3.

A wide range of isotopes including ^99m^Tc, ^45^Ti, ^89^Zr, ^131^I and ^18^F have been successfully used to radiolabel MSNs (Table 2). Before radiolabeling, ^99m^Tc is reduced to a lower oxidation state to facilitate its binding to ligands and the synthesis of radiopharmaceuticals. Several reducing agents have been investigated, but stannous chloride is the preferred choice because of its favorable stability and low toxicity [21].

## 3. Radiolabeling

Radiolabeled nanomaterials exist in three forms: chelator-free materials, those with bifunctional chelating agents (BFCs), and those prepared by encapsulation radiolabeling. Advantages and limitations of these strategies are presented in Table 3. All three can be applied to silica nanoparticles with their large numbers of silanol groups (–Si–O–) located on the surface or within the mesoporous architecture of silica. These can serve as intrinsic coordination sites for chelator-free radiolabeling or can be easily functionalized with suitable chelators. Stable silica nanoparticles can be prepared using direct labeling, which does not use chelators or spacers, or indirect labeling, which requires bifunctional chelators or spacers. Each radiometal requires the selection of a suitable linker, and the radioisotope can be encapsulated during or after synthesis. The choice of appropriate labeling affects the physical and chemical properties of nanoparticles, which can be evaluated by measuring size, zeta potential and surface properties; however, these properties should not significantly change during radiolabeling [46].

### 3.1. Chelator-Free Radiolabeling

In direct radiolabeling, radionuclides are covalently or coordinatively bound to functional groups on the nanoparticle surface. By employing an appropriate nanoplatform–radioisotope combination, intrinsic labeling can eliminate challenges related to chelator instability and labor-intensive chelator functionalization [47]. The successful preparation of intrinsically radiolabeled nanoparticles relies on strong interactions between carefully selected nanoparticle platforms and radionuclides.

Porthilo et al. [28] developed, characterized and tested in vivo a smart delivery system, based on radiolabeled magnetic core mesoporous silica doped with trastuzumab as an intralesional nanodrug for breast cancer imaging and possible therapy. Radiolabeling was conducted without the use of a chelator. To investigate the optimum conditions, radiolabeling was tested with different concentrations of reducing agent (100, 200, 500 micrograms of SnCl_2_). An interesting finding of the study was that with increasing concentration of the reducing agent, higher chemical purity values were achieved. Biodistribution following systemic administration showed substantial nanoparticle uptake by the lungs (~30%). This could be explained by retro-orbital administration, which introduces nanoparticles directly into the systemic circulation, leading to first-pass passage through the lungs and resulting in pronounced pulmonary accumulation. Another theory suggests that this could be due to the high affinity of silica for the lungs [48]. Hepatic uptake was around 12.85%, which is, in turn, a characteristic phenomenon observed for various nanoparticles and may be explained by recognition of nanoparticles by phagocyte surface receptors and/or recognition of nanomaterials via protein adsorption [49]. However, in case of intralesional route, the highest amount of radiolabeled nanoparticles (97.37%) remains in the tumor. The presence of trastuzumab both on the surface and within the magnetic core of mesoporous silica enhances antigen–antibody binding affinity. These interactions are mediated by hydrogen bonding, hydrophobic interactions, and van der Waals forces. Binding to the cell surface induces membrane invagination, followed by scission of the plasma membrane, resulting in the formation of endocytic vesicles that facilitate intracellular transport of the nanoparticles into the cytoplasm.

Chen et al. [30] developed a radiolabeling method that allows efficient incorporation of the ^45^Ti isotope using MSN. To evaluate the feasibility of employing ^45^Ti-labeled MSNs for in vivo tumor-targeted PET imaging, the nanoparticles were first functionalized with –NH_2_ groups, subsequently labeled with ^45^Ti under controlled conditions (pH 7–8, 75 °C), and finally subjected to a standard PEGylation procedure ([^45^Ti]MSN-PEG5k). Intrinsic ^45^Ti labeling was achieved via strong coordination between ^45^Ti, a hard Lewis acid, and oxygen donor atoms acting as hard Lewis bases, specifically the deprotonated silanol groups (–Si–O–) located on both the external surface and within the mesoporous channels of the MSNs. Approximately 80% labeling yield was achieved within 30 min of incubating. It is noteworthy that the labeling yield further increased to ~90% after an additional 30 min incubation. The PET scan was used to demonstrate differences in biological distribution 30 min after the injection. Mice administered [45Ti]MSN-PEG5k exhibited a pharmacokinetic profile, characterized by predominant accumulation in the liver (44% ID/g). The values for heart and tumor uptake were 7.4 ± 1.8% ID/g and 4.6 ± 1.4% ID respectively.

^89^Zr possesses a favorable half-life (t_1/2_ = 78.4 h) and a relatively low average positron energy (β^+^avg = 395.5 keV), making it well-suited for prolonged in vivo tracking of nanoparticles, minimizing nonspecific background tissue uptake, and providing advantageous dosimetric properties [50]. Currently, the most commonly used ligand for labeling ^89^Zr^4+^ is desferrioxamine B, a ligand containing three hydroxamic groups. Nevertheless, substantial bone uptake of ^89^Zr (3–15% ID/g) has been consistently reported in various ^89^Zr-DFO-mAb studies up to 7 days post-injection (p.i.), suggesting the gradual release of free ^89^Zr and the limited stability DFO-based ^89^Zr labeling [51,52]. Significant bone uptake of ^89^Zr reflects the osteophilic nature of the radionuclide. Assessment of bone uptake over time can serve as a valuable indicator of the in vivo stability of ^89^Zr-labeled nanoparticles.

A study of chelator-free zirconium-89-labeled mesoporous silica nanoparticles [34] found a higher concentration of MSN and high incubation temperature to give a higher labeling yield. The study demonstrated the significant role of deprotonated silanol groups in the effective labeling of ^89^Zr on MSNs without the use of chelating agents. In this case, Zr^4+^ is a hard Lewis acid and the deprotonated silanol groups (–Si–O–) are hard Lewis bases. Silanol groups (–Si–OH) undergo complete deprotonation to form –Si–O^−^ species when the pH of the aqueous environment exceeds the isoelectric point of silica, which is typically reported to lie in the pH range of 2–3. High concentration and high incubation temperature gave higher labeling yield. Deprotonated silanol groups play a vital role in chelator-free labeling of ^89^Zr to MSN. TEM images of ^89^Zr-MSN (acquired after radioactive decay) revealed no discernible changes in morphology following ^89^Zr labeling. Maximum intensity projection (MIP) images demonstrated predominant accumulation in the liver and spleen, with no evident bone uptake within 3 h post-injection (p.i.). Mice administered ^89^Zr-MSN exhibited exceptionally high in vivo stability over a 3-week period, with bone uptake remaining below 1% ID/g at 7 days post-injection (p.i.). The comparatively lower, though non-negligible, bone uptake observed for ^89^Zr-MSN may be ascribed to the partial dissociation of ^89^Zr^4+^ ions localized on the external surface, which are not necessarily confined within the mesoporous channels of the MSNs. Considering that the coordination of ^89^Zr^4+^ ions with silanol groups is expected to be comparatively weaker at the external surface—where the local density of silanol functionalities is lower than within the confined mesoporous channels—the surface-bound ^89^Zr^4+^ species are likely to exhibit increased lability. 

Another study [37] described the cellular uptake and in vivo photodynamic therapy potential of Zn phthalocyanine-loaded mesoporous silica nanoparticles (MSNP5) that were evaluated in pancreatic cancer cells. Both MSNP5 and the cetuximab antibody (C225) were radiolabeled with the ^131^I radionuclide using the Iodogen method [53]. It was determined that the yield of radiolabeling increased significantly with increasing reaction time. In vitro studies comparing radiolabeled MSNP5 and cetuximab demonstrated that ^131^I-MSNP5 exhibited approximately tenfold higher cellular uptake than ^131^I-C225. Cellular uptake assays revealed that ^131^I-MSNP5 accumulation reached 43.9 ± 3.8%, 41.8 ± 0.2%, and 37.9 ± 1.3% in PANC-1, AsPC-1, and MIA PaCa-2 cells, respectively. These findings suggest that ^131^I-labeled MSNP5 represents a promising multifunctional platform for photodynamic therapy, with additional potential for radionuclide-based imaging.

Fazaeli et al. [42] attached the radionuclide ^68^Ga to pyridine-functionalized mesoporous silica (MCM-41) to develop an image-guided drug delivery system for diagnostic purposes ([^68^Ga]-Py-Butyl@MCM-41). The tumor-targeting efficiency and pharmacokinetic profile of the developed nanocomposite were evaluated for the first time in mice bearing fibrosarcoma. The aim of attaching the gallium-68 radionuclide to mesoporous silica MCM-41 was to combine the features of pyridine-functionalized silica—such as low mass, large surface area, and cytotoxic effects on cancer cells—with the diagnostic capabilities provided by gallium-68. Because of the large surface area of the synthesized nanoparticles (638 m^2^/g), the required quantity of particles was significantly reduced compared to other polymer-based systems [43]. Owing to their size and the uptake by the mononuclear phagocyte system, most of the radioactivity detected one hour post-injection was localized in the spleen, liver, and lungs, as well as in the tumor. The high stability of the nanocomposite in aqueous media, conferred by surface hydroxyl functionalities on the silica framework, together with the moderate lipophilicity of the compound (log P = 0.68), resulted in a uniform and homogeneous distribution of the radiolabeled material within the matrix.

### 3.2. Radiolabeling with BFCs

Bifunctional chelating agents are necessary for radiolabeling biomolecules with a metallic radionuclide.

#### 3.2.1. DTPA/DTPA Analogs

Diethylenetriaminepentaacetate (DTPA, also known as pentetic acid) is a synthetic polyaminocarboxylic acid with eight coordinate bond-forming sites that can sequester metal ions and form highly stable DTPA–metal ion complexes. DTPA has been a commonly used chelator in nuclear medicine applications for radionuclides such as ^111^In, ^212^Bi, ^90^Y, ^99m^Tc, ^67,68^Ga and is one of the earliest chelators used in the development of FDA-approved radiopharmaceuticals [54]. It is worth noting that pentetic acid can bind directly to other trace metals in the body known to cause deficiencies [55].

DTPA analogs possess extremely high radiolabeling efficiency as BFCs under mild conditions. p-SCN-Bn-DTPA (S-2-(4-Isothiocyanatobenzyl)-diethylenetriamine pentaacetic acid) is used for the radiolabeling of silica nanoparticles [56].

Gao et al. [23] describe the sequential reaction of manganese oxide-based mesoporous silica nanoparticles (MnOx-MSNs) with p-SCN-Bn-DTPA (∼500 nmol); the product was intended as a radiolabeling mediator for use in SPECT-MRI dual-modal imaging. The reaction was performed at pH = 9.0. The radiolabeling yield was as high as 99.1 ± 0.6%. The authors in the study made use of the fact that the pH value of the tumor microenvironment is lower than that for normal tissues due to its up-regulated glycolytic metabolism [57]. Therefore, in the mildly acidic tumor microenvironment, MnOx nanoparticles can convert into Mn^2+^, leading to an increase in the r1 relaxivity and enabling more effective T1-weighted MRI [58]. In fact, MnOx nanoparticles within tumor tissue can be converted to Mn^2+^, which facilitates the probing of the tumor microenvironment and enables early-stage tumor detection. In vivo MRI and SPECT imaging in tumor-bearing mice demonstrated a clear enhancement of tumor visualization, combining MRI’s high spatial resolution with the exceptional sensitivity of SPECT. As reported in the literature, both high radiochemical yield and robust in vivo stability are critical parameters for the development of an effective new radiopharmaceutical [59]. The radiochemical yield of ^99m^Tc-Mn-MSNs-PEG was determined using TLC analysis, revealing a high mean labeling efficiency of 99.1 ± 0.6% (*n* = 3). Importantly, the radiochemical stability of ^99m^Tc-Mn-MSNs-PEG remained above 95% after 24 h incubation in 50% fetal bovine serum at 37 °C.

In another study, conducted by Branco de Barros et al. [25], MSNs functionalized with 3-aminopropyltriethoxysilane (APTES) were then anchored with DTPA and then radiolabeled with technetium-99m, showing high radiochemical yields (98.3 ± 0.7%) and high radiolabeled stability. APTES can bind to a silica surface in three ways: tridentate, bidentate, or monodentate. This is possible due to the presence of triethoxysilane groups on the surface of this ligand. The experimental C:N ratio for the MSN/APTES compound was 5, suggesting that the APTES is predominantly condensed in the bidentate form. The rise in carbon and nitrogen levels after introducing DTPA groups suggests that this functional group has been effectively attached to the surface of MSN-APTES. In vivo experiment results revealed a high accumulation in the liver (20.05 ± 0.55% ID/g), as anticipated due to phagocytic activity. However, the particles also exhibited considerable uptake in the lungs (40%), reflected by a high lung-to-non-target tissue ratio.

Içhedef et al. [22] performed a study with an MSN system radiolabeled with ^99m^Tc. DTPA was used as a chelating agent. The MSN carrier system was loaded with methotrexate (MTX), an anticancer drug used in the treatment of breast cancer, with DTPA used as a BFC. In total, 30 mg of MSN-APTES was dispersed in 5 mL of dimethylformamide (DMF), after which 130 mg of DTPA was added. The reaction was performed at 80 °C overnight. The results show high efficiency and stability. MSN particles exhibit a negative zeta potential of −5.31 mV, attributable to deprotonated silanol groups (Si–OH Si–O^−^) present on the silica surface. The zeta potential of MSN-DTPA was determined to be −27.1 mV. The substantial decrease in surface charge following DTPA functionalization can be attributed to the presence of negatively charged carboxylate (COO^−^) groups of DTPA, which are grafted onto the MSN surface. The surface charge of nanoparticles plays a critical role in colloidal stability by reducing aggregation through electrostatic repulsion, and it can also influence their behavior in biological environments in vivo [25]. The methotrexate-loaded MSN particles exhibited a high radiolabeling efficiency, with an average value of 92.20 ± 0.83% (*n* = 5). To evaluate the in vitro stability of ^99m^Tc-MTX-MSNs, the radiolabeling efficiency was monitored over a 24 h period following the labeling reaction. The results demonstrated that the radiolabeled nanoparticles remained relatively stable in saline during the initial phase of incubation. Approximately 75% stability was retained after 240 min, while a gradual decline was observed over time, with the radiolabeling efficiency decreasing to 65% at 24 h.

Andrade et al. [27] synthesized particles made of mesoporous silica (SBA-16) and silica containing a calcium phosphate component (SBA-16/HA). The particles were functionalized with APTES by post-synthesis grafting and loaded with the antibiotic ciprofloxacin. Following this, DTPA was anchored to enable chelating of technetium-99m. They obtained two complexes: ^99m^Tc-DTPA-SBA-16APTES and ^99m^Tc-DTPA-SBA-16/HAAPTES. Thin-layer chromatography (TLC) was employed to estimate the radiochemical yields of ^99m^Tc-DTPA-SBA-16APTES and ^99m^Tc-DTPA-SBA-16/HAAPTES. The findings indicated that the average radiochemical yield was 98.10 ± 1.02% for ^99m^Tc-DTPA-SBA-16APTES and 99.74 ± 0.08% for ^99m^Tc-DTPA-SBA-16/HAAPTES (*n* = 6). Radiochemical purity above 90% is recommended for radiopharmaceuticals, which was achieved for both ^99m^ Tc-DTPA-SBA-16APTES and ^99m^Tc-DTPA-SBA-16/HAAPTES. As expected, both complexes were uptaken by the liver and spleen. Nanostructures are typically identified by the mononuclear phagocyte system, particularly by macrophages located in these organs [60]. Both radiocomplexes showed minimal accumulation in the thyroid and stomach, supporting the in vitro stability findings, as free technetium-99m is known to preferentially localize in these organs [61]. Regarding to bone uptake, a higher uptake for ^99m^Tc-DTPA-SBA-16/HAAPTES was noticed when compared to ^99m^Tc-DTPA-SBA-16APTES, at 1 and 4h post-injection.

#### 3.2.2. Deferoxamine (Desferrioxamine/Desferal/DFO/DFOA/DFOM)

DFO is a natural product isolated from Streptomyces pilosus. As it readily forms iron complexes, it is used as a chelating agent to treat iron or aluminum toxicity and some blood transfusion-dependent anemias.

In one study, amine-grafted MSNs were reacted with the DFO analog p-SCN-deferoxamine (DFO) in dimethyl sulfoxide (DMSO) [33]. The levels of reacted DFO in the supernatant before and after conjugation were determined by UV-Vis absorption (281 nm). Radiolabeling with ^89^Zr^4+^ was performed by covalent attachment of DFO. As expected for silica under the investigated conditions, the non-functionalized particles exhibited a negative zeta potential. Following amine functionalization, the surface charge shifted to positive values. Subsequent covalent conjugation of DFO, serving as a chelator for ^89^Zr^4+^, resulted in a reduction in the absolute magnitude of the zeta potential, indicating partial neutralization of surface charge. Tumor uptake of PEGylated nanoparticles was 3.8% ID/g in PC-3 and 8.6% ID/g in LNCaP C4-2 tumors. The presence of MSNs in the lungs, heart, and brain was primarily attributed to the blood pool within these organs rather than active uptake mechanisms. In contrast, accumulation in tumors, liver, and spleen resulted from active uptake processes.

#### 3.2.3. 2,3,5,6-Tetrafluorophenyl 6-[^18^F]-Fluoronicotinate ([^18^F]FPyTFP)

Another prosthetic group used for radiolabeling is [^18^F]FPyTFP. [^18^F]FPyTFP is developed from the precursor N,N,N-trimethyl-5-[(2,3,5,6-tetrafluorophenoxy)carbonyl] pyridin-2-aminium trifluoromethanesulfonate [62].

Simó et al. [38] tested radiolabeled mesoporous silica-based urease-powered nanobots in an orthotopic mouse model of bladder cancer. Briefly, 450 nm sized MSNPs were synthesized using a modified Stöber method [63] and then radiolabeled with the positron emitter ^18^F to enable quantification by PET imaging. Radiofluorination was carried out via a labeled prosthetic group (the 2,3,5,6-tetrafluorophenyl ester of 6-[^18^F]fluoronicotinic acid, [^18^F]F-PyTFP), which was conjugated to the amino groups present on the enzyme [64]. Radiochemical purity after purification was ≥99%, as determined by radio thin-layer chromatography (radio-TLC). An orthotopic syngeneic mouse model was employed to assess the capacity of the nanobots to localize to the tumor site. Subsequent histopathological analysis of the tumors, performed using hematoxylin–eosin staining after PET imaging, verified the presence of a solid neoplastic mass in tumor-bearing mice, situated beneath the lamina propria and exhibiting growth toward the bladder lumen. PET imaging, using computed tomography as an anatomical reference, was employed to investigate the accumulation of nanobots within the tumor. PET imaging demonstrated an approximately eightfold enhancement in nanobot accumulation within the tumor tissue. In tumor-bearing animals, PET imaging demonstrated that the radioactive signals corresponded spatially with tumor locations as determined by MRI. The therapy involved the intravenous administration of iodine-labeled nanobots. As a result of the conducted studies, a 90% reduction in tumor size was observed. This confirmed the validity and efficacy of the applied nanobots as drug delivery systems in bladder cancer therapy.

#### 3.2.4. N-Succinimidyl-4-Fluorobenzoate (SFB)

**Scheme 1:**
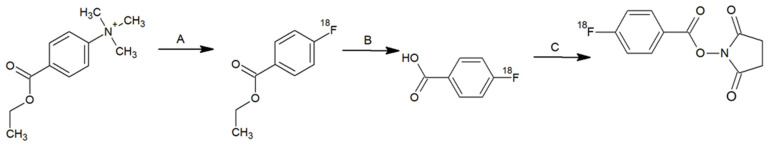
Radiosynthesis of [^18^F]SFB reported by Wester et al. [66]. A: K+, Kryptofix 2.2.2, ^18^F-, CH_3_CN, 90 °C; B: 120 °C; C: TSTU 90 °C.

Another radiolabel, [^18^F]SFB was first synthesized in 1992 [65]. Briefly, [^18^F]fluoride was incorporated into 4-formyl-N,N,N-trimethylanilinium-triflate, and the obtained product was oxidized to 4-[^18^F]fluorobenzoic acid; this was then activated by dicyclohexylcarbodiimide (DCC) treatment to produce [^18^F]SFB. The procedure was subsequently modified by Wester et al. [66] by replacing the benzaldehyde precursor with trimethylammonium)benzoate, and using O-(N-succinimidyl-N,N,N′,N′-tetramethyluronium tetrafluoroborate (TSTU) to convert the benzoic acid intermediate to [^18^F]SFB (Scheme 1).

Radiolabeling nanostructures with N-succinimidyl 4-[^18^F]fluorobenzoate [^18^F]SFB allows them to be used as diagnostic agents in PET.

Rojas et al. [39] anchored an ^18^F positron emission isotope to mesoporous silica nanoparticles (30–130 nm) by functionalization with amino groups followed by reaction with ^18^F-SFB. Briefly, ω-aminopropyl-functionalized silica nanoparticles (NH_2_SiNPs) were stirred at room temperature for 45 min in a mixture of [^18^F]SFB, DMSO and sodium citrate buffer (pH 11). The purpose of preparing modified MSiNPs with aminopropyl groups on their surface was to enable the covalent attachment of the ^18^F isotope through the succinimidyl functionalization of the ^18^F-SFB synthon. Thus, the free amino groups of NH_2_-MSiNPs were conjugated with succinimidyl groups to incorporate the isotopically labeled 4-(^18^F)fluorobenzoyl moiety via ^18^F-SFB, enabling tracking of the biodistribution of ^18^F-MSiNPs. This approach was selected based on the well-established high reactivity of succinimidyl compounds toward amino groups, which has been widely utilized in various radiolabeling procedures [67]. This novel MSiNP labeling approach is more user-friendly and straightforward compared with previous methods, which involved direct ^18^F labeling of silicon atoms under harsh reaction conditions [68]. The radioconjugates were then administered intravenously in murine models and their in vivo biodistribution determined using PET. It was reported that nanoparticles accumulated in the organs of the reticuloendothelial system by phagocytosis and in the capillary network of the lungs by vascular trapping, and were excreted into bile and urine. As a consequence of their excretion, their vascular trapping and the active phagocytosis of the reticuloendothelial system, the plasmatic bioavailability of ^18^F-MSiNPs was low. Although the in vivo and ex vivo biodistribution profiles were highly comparable, the in vivo measurements consistently yielded lower concentrations across all organs relative to ex vivo analyses. This discrepancy was particularly pronounced in smaller organs, such as the spleen and lungs, suggesting that it may be attributable to the partial volume effect. This represents a well-recognized limitation of PET imaging in pharmacokinetic studies in small laboratory animals, arising from the limited spatial resolution of the technique, which can lead to an underestimation of the true radioactivity concentration.

#### 3.2.5. NOTA/NOTA Analogs

The macrocyclic chelator NOTA (1,4,7-triazacyclononane-1,4,7-triacetic acid) and its derivatives offer fast and efficient radiolabeling and in vivo stability, making them particularly suitable for incorporating certain isotopes [30]. However, while NOTA represents an attractive framework for constructing PET imaging tools, it is not suitable for creating targeted PET probes because of its limited bifunctionality.

While NOTA possesses three carboxyl groups, the conjugation of a carboxyl group with a targeting vector may reduce its overall coordinating potential. Despite this, various derivatives allow vector conjugation while retaining chelation capability: S-2-(4-isothiocyanatobenzyl)-1,4,7-triazacyclononane-1,4,7-triaceticacid (p-SCN-Bn-NOTA), 1,4,7-triazacyclononane-1-succinic acid-4,7-diacetic acid (NODASA) and 2-(4,7-bis-(carboxymethyl)-1,4,7-triazonan-1-yl)pentanedioic acid (NODAGA) [69].

**Figure 4:**
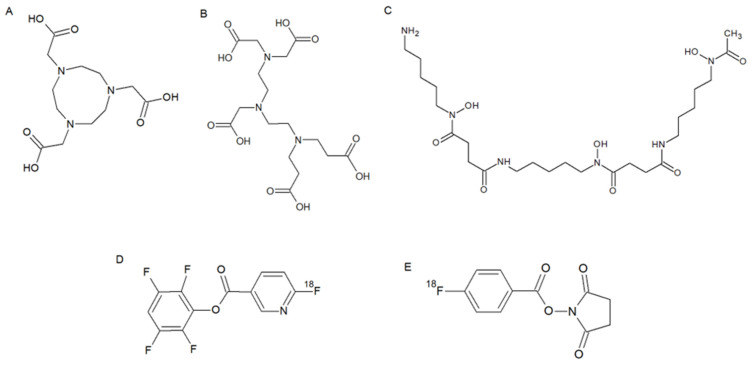
Chemical structures of BFC: (**A**)—NOTA; (**B**)—DTPA; (**C**)—DFOM; (**D**)—[^18^F]FPyTFP; (**E**)—SFB.

NOTA has been used to create ^64^Cu-NOTA-mSiO_2_-PEG-TRC105, based on the development of a functionalized mSiO_2_ nanoparticle and ^64^Cu-labeling [41]. Monodisperse mSiO_2_ nanoparticles were prepared via a well-established soft-template synthesis [70] and subsequently functionalized with TRC105 antibody (specific for CD105/endoglin) and (S)-2-(4-isothiocyanatobenzyl)-1,4,7-triazacyclononane-1,4,7-triacetic acid (p-SCN-Bn-NOTA) through polyethylene glycol (PEG) spacers. The construct was then labeled with ^64^Cu, yielding the ^64^Cu-NOTA-mSiO_2_-PEG-TRC105 nanoconjugate. Serum stability assays confirmed that over 90% of the radioactivity remained nanoparticle-bound after 48 h. This high radiolabel stability ensures that the PET signal reliably reflects the biodistribution of ^64^Cu-labeled mSiO_2_ nanoparticles. PET imaging and biodistribution analyses demonstrated that the liver, intestines, kidneys, spleen, and tumor represent the main sites of uptake for ^64^Cu-NOTA-mSiO_2_-PEG-TRC105. The pronounced hepatic accumulation at 5 and 48 h post-injection suggests that clearance of ^64^Cu-NOTA-mSiO_2_-PEG-TRC105 primarily occurs via the hepatobiliary pathway. The gradual reduction in liver uptake over time is likely attributable to the slow excretion of both ^64^Cu-NOTA-mSiO_2_-PEG and ^64^Cu-NOTA-mSiO_2_-PEG-TRC105 into the intestinal tract, which is further supported by biodistribution data showing notable intestinal accumulation. On Figure 4, the chemical structures of five BFCs are presented: NOTA, DTPA, DFOM, [^18^F]FPyTFP, and SFB.

**Table 3:** Advantages and limitations of chelator-based and chelator-free radiolabeling approaches.

Types of Radiolabeling	Advantages	Limitations	Ref.
Chelator-free radiolabeling	Simple synthesis. No additional chelating agents. Highly desirable for whole-body imaging. Maintenance of the intrinsic surface properties of MSNs. For radionuclides such as ^89^Zr, strong interaction with oxygen in the SiO_2_ matrix may provide greater stability than some chelate complexes.	Limited number of relevant radionuclides. Stability depends on the surface chemistry. Difficulty in developing a universally applicable method.	[5,34]
Chelator-based radiolabeling	Well-understood chemistry and high reproducibility of procedures. Easier standardization and compliance with GMP requirements. Numerous examples of clinical applications of chelating agents. High stability and versatility. Flexibility in design.	The diverse coordination chemistry of radioisotopes prevents the use of a universal chelator. Transchelation by endogenous proteins or chelator detachment can result in radiolabel loss and misleading biodistribution data. Need for additional surface modification of MSNs. Increasing the complexity of the synthesis. Radionuclide release from unstable complexes can distort biodistribution imaging.	[71,72,73]
Encapsulation radiolabeling	High stability in vivo. The signal does indeed reflect the fate of the nanoparticle. The risk of nuclide release is minimal.	Not suitable for the labeling of nanoparticles conjugated with biological molecules. Difficulties in scaling up production. The need to work with radioactive material as early as the synthesis stage.	[74]

Most medically relevant radioisotopes form stable coordination complexes with chelators containing oxygen, sulfur, or nitrogen donor atoms [75]. Accordingly, chelator-free intrinsic radiolabeling may be enabled by nanoparticles containing appropriately arranged oxygen donor atoms. Among suitable materials, the silica nanoparticle stands out because of its facile synthesis and proven biomedical applicability.

**Figure 5:**
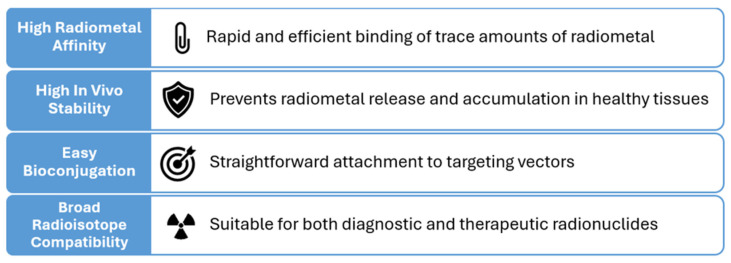
Key features for an ideal chelator.

Different systems require the use of different chelators for radiolabeling. However, when selecting an appropriate chelator, it is important to consider properties that enable efficient labeling and ensure safety. The selected features are summarized in Figure 5.

## 4. Imaging Modalities for the In Vivo Valuation of Radiolabeled MSNs

Radiolabeled nanoparticles can provide information on biological processes, including tumor receptor expression and enzymatic activity, through the detection of positron and gamma radiation emitted by the radionuclide using PET or SPECT imaging systems.

Medical imaging techniques are those used to create visual representations of tissues and organs within the human body to observe both normal and pathological anatomical and physiological conditions. These modalities include X-rays, computed tomography (CT), positron emission tomography (PET), magnetic resonance imaging (MRI), single-photon emission computed tomography (SPECT), digital mammography, and diagnostic ultrasound [76]. The first medical imaging technique was X-ray imaging, developed by Wilhelm Conrad Roentgen in November 1895. The generation of more powerful X-ray emitters resulted in the creation of fluoroscopy, which enables real-time imaging for the diagnosis of patient abnormalities.

At present, the most widely used non-invasive imaging modalities for disease diagnosis and assessment of treatment response include magnetic resonance imaging (MRI), computed tomography (CT), positron emission tomography (PET)/single-photon emission computed tomography (SPECT), and ultrasonography (US). The combination of multiple imaging modalities in hybrid imaging offers complementary and comprehensive information, thereby addressing the inherent limitations of individual imaging techniques [77].

Fluoroscopy has in turn undergone significant technological advancements, resulting in the development of computed tomography (CT) also known as X-ray CT, which provides high-resolution, cross-sectional imaging for more precise anatomical and pathological assessment. The first scanner prototype was created in the 1969s, by Hounsfield [78]. Nowadays, CT scan is used for the diagnosis of various conditions.

Among these imaging modalities, PET and SPECT are classified as nuclear medicine. SPECT/PET and MRI represent the two most widely applied clinical imaging modalities, as SPECT/PET is characterized by excellent sensitivity and limited spatial resolution, while MRI offers high spatial resolution but comparatively lower sensitivity [79]. They are distinct from CT, and the approaches are often combined for functional and anatomical imaging.

### 4.1. PET Imaging of Radiolabeled MSNs

Positron emission tomography (PET) is a non-invasive, quantitative imaging modality that requires the incorporation of positron-emitting radionuclides. Since the PET signal directly reports the biodistribution of the radionuclide rather than that of the delivery vehicle or therapeutic cargo, its application in image-guided drug delivery is fundamentally dependent on the robustness of the radiolabeling strategy and the in vivo stability of the labeled construct. PET scanning can be employed to detect inter alia atherosclerosis, aging-related changes, cancer and schizophrenia [80]. Briefly, the radioisotope releases a positron as a result of positive beta decay. As the positron moves through tissue, it gradually loses energy due to interactions with surrounding matter. After traveling a short distance, it undergoes annihilation with an electron. This process produces two gamma photons, each with an energy of 511 keV. Provided that these photons (gamma rays) do not undergo further interactions, they will travel in exactly opposite directions [81]. These are then detected by the PET system.

Ferreira et al. [82] reported the use of newly developed ultrasmall porous silica nanoparticles (UPSNs) with improved in vivo pharmacokinetics, including high accumulation in target tissues (12% ID/g in tumors) and reduced uptake by reticuloendothelial system (RES) organs. UPSNs were conjugated with the isotopic pair ^90/86^Y, enabling both non-invasive imaging as well as internal radiotherapy. ^86^Y-DOTA-UPSN provided high contrast against the tumor and prolonged circulation time in PET imaging studies.

Several studies have demonstrated the suitability of PET for tracking MSN-based nanocarriers after systemic administration. For instance, Chen et al. [30] employed ^45^Ti-labeled mesoporous silica nanoparticles to investigate their biodistribution and pharmacokinetic profile, demonstrating preferential accumulation in the liver and spleen. Similarly, Goel et al. [32] developed ^89^Zr-labeled dendritic MSNs conjugated with TRC105 antibodies, which enabled long-term in vivo tracking and revealed efficient tumor targeting. Further studies involving PEGylated ^89^Zr-MSNs [33] showed that PET imaging could differentiate nanoparticle uptake between prostate cancer models, highlighting the importance of surface modification for enhancing tumor accumulation.

PET has also been used to evaluate other radionuclide-labeled silica nanoplatforms. ^18^F-labeled mesoporous silica nanoparticles [39] enabled whole-body imaging and revealed significant accumulation within reticuloendothelial organs, whereas ^64^Cu-NOTA-mSiO_2_-PEG-TRC105 nanoparticles [41] exhibited high tumor uptake and favorable pharmacokinetic properties. Moreover, PET imaging has proven useful in monitoring the in vivo behavior of arsenic-loaded MSNs [38] and ultra-porous silica nanoparticles labeled with ^86^Y [82] thereby supporting the development of MSN-based theranostic systems. The possibility of combining PET with CT or MRI provides additional anatomical information and improves the localization of nanoparticle accumulation.

Radioarsenic isotopes possess significant theranostic potential. Diagnostic ^72^As can be produced either using existing medical cyclotrons equipped with solid-target capabilities or via a ^72^Se/^72^As generator system [83]. The radionuclide ^77^As for therapeutic applications can be generated in significant quantities using nuclear fission reactors under no-carrier-added conditions [84]. The compounds have been found to enable PET imaging aimed at guiding the delivery of the chemotherapeutic agent arsenic trioxide (ATO) [38]. Also, due to the strong affinity between arsenic and surface thiol groups, thiolated MSNs act as highly stable carriers for As(III). Radiolabeling was achieved by incubating thiolated nanoparticles with no-carrier-added As(OH)_3_, achieving labeling efficiencies of 40 to 90%, depending on nanoparticle type and thiol density. The radioarsenic labeling of thiol-modified MSN was shown to be pH-dependent with a higher labeling pH exhibiting significantly improved labeling yield. Higher labeling temperature (80 °C) was demonstrated to be unfavorable for labeling. It was hypothesized that this was due to the rapid oxidation of *As(III) oxidation to *As(V) at higher temperature. *As(V) does not label thiol-modified MSN. These findings obtained with no-carrier-added (n.c.a.) radioarsenic are consistent with recent studies on radioarsenic and with the well-established chemical behavior of bulk trivalent and pentavalent arsenic. As recently reviewed in the context of protein interactions, As(III) exhibits strong affinity for thiol groups, whereas As(V) shows negligible binding [85]. The in vitro stability of *As-labeled MSNs (3 nm pores) was assessed by incubation in whole mouse serum for 18 h at room temperature, followed by four days at 37 °C. In vivo stability was evaluated through serial PET imaging in mice after injection of free *As, *As-dSiO_2_, As-MSN (3 nm), and As-MSN (5 nm). PET studies confirmed prolonged retention of radioarsenic, lasting from days to weeks, and that increasing mesoporosity enhances in vivo stability, likely due to higher surface thiol density. Compared to protein-based radiolabeling, this strategy offers superior radiostability and allows high ATO loading (20 mg/g), supporting its potential for PET-guided, arsenic-based theranostic applications [34].

A multifunctional PDI-organosilica nanoplatform (HMPDINs) was developed by in situ PDI incorporation during HMON synthesis [86]; the product has achieved enhanced FL/PA signals through silica shell shielding. The system can be subjected to ^64^Cu labeling and SN38 loading through in situ polymer growth, thus enabling trimodal FL/PA/PET imaging and laser-triggered drug release. The platform combines photothermal therapy with SN38 chemotherapy, demonstrates synergistic antitumor effects, and offers a promising strategy for precise, multimodal cancer theranostics. Yang et al. [86] reported a special “in situ framework growth” method. It enables the synthesis of novel phototheranostic hollow mesoporous nanoparticles by innovative hybridization of perylene diimide (PDI) within the framework of hollow mesoporous organosilica (HMO). The isotope ^64^Cu was used to chelate the organosilica shell. Combining PDI with HMO gave the phototheranostic silica nanoparticles (HMPDINs) significantly stronger fluorescence and photoacoustic responses, enabling improved performance in both fluorescence and photoacoustic imaging. A total of 100 μCi of ^64^Cu@HMPDINs@TP was administered intravenously to mice with U87MG tumors. PET image intensity data were derived by defining regions of interest in key organs and tumors on whole-body coronal images that had been corrected for radiation distribution.

Consequently, PET remains one of the most powerful tools for the preclinical and clinical evaluation of radiolabeled mesoporous silica nanoparticles.

### 4.2. SPECT Imaging of Radiolabeled MSNs

SPECT is a diagnostic method used in nuclear medicine. It allows for the visualization of functional and metabolic processes in the human body. SPECT is based on the detection of γ radiation emitted by single-photon-emitting radionuclides. A detector is used to capture γ-rays and convert them into electrical signals. These electrical signals are then processed by a computer system to generate detailed tomographic reconstructions and whole-body images, which allow for the visualization of radionuclide biodistribution throughout the organism. In SPECT imaging, organs exhibiting higher uptake of the radiotracer are visualized as regions of increased signal intensity. Alterations in the distribution or absorption pattern are reflected by variations in the brightness of these areas, thereby enabling the identification of various pathological conditions [87].

Because nanoparticles themselves do not emit positrons, PET/SPECT imaging relies exclusively on signals originating from the attached radioisotopes, which are commonly linked to the nanoparticle surface through a chelator. However, free radioisotopes or chelator-bound isotopes often exhibit pharmacokinetic behaviors that differ from those of the nanoparticle. Consequently, in vivo dissociation of the radioisotope from the nanocarrier may result in misleading PET/SPECT data and inaccurate interpretation of nanoparticle biodistribution. The effectiveness of radiolabeled nanoparticles depends strongly on the careful choice of radionuclides, nanocarriers, and targeting ligands. Diagnostic radionuclides such as ^43m^Tc, ^64^Cu, ^18^F, and ^111^In enable real-time tumor and metastasis imaging via SPECT or PET [88]. In contrast, therapeutic radionuclides including ^177^Lu, ^90^Y, ^131^I, and ^225^Ac deliver cytotoxic radiation to tumor tissues [89].

SPECT radionuclides (^99m^Tc, ^123^I, ^201^Tl, ^111^In, ^133^Xe) are gamma emitters used for three-dimensional functional imaging and lymphatic metastasis tracking, while PET radionuclides (^18^F, ^68^Ga, ^82^Rb, ^11^C, ^13^N, ^15^O, ^64^Cu, ^89^Zr) are positron emitters that enable high-resolution visualization of tumor metabolism and receptor expression. Compared with PET, SPECT using γ-emitters such as ^99m^Tc and ^111^In offers longer imaging windows, reduced cost, and greater radionuclide availability [81].

Manganese oxide-coated mesoporous silica nanoparticles labeled with ^99m^Tc [23] were successfully applied for dual SPECT/MRI and demonstrated excellent radiochemical stability together with enhanced tumor visualization, as did ^99m^Tc-MSN-APTES-DTPA nanoparticles [24]. Other studies involving ^99m^Tc-functionalized MSNs [27,28] confirmed the applicability of SPECT imaging for evaluating the organ distribution and in vivo behavior of silica-based nanocarriers. In addition, ^177^Lu-containing mesoporous silica nanoparticles [44] have demonstrated considerable potential for combined imaging and radionuclide therapy. Although SPECT generally exhibits lower spatial resolution and quantitative accuracy than PET, its high sensitivity, lower cost, and compatibility with hybrid SPECT/CT systems make it an attractive imaging modality for the investigation of MSN biodistribution and therapeutic performance.

### 4.3. MRI-Based MSN Systems

Magnetic resonance imaging (MRI) is founded on the principles of nuclear magnetic resonance (NMR). It is based on the ability of atomic nuclei to absorb and emit energy when exposed to a magnetic field. In this technique, hydrogen nuclei serve as the main source of signal due to their high presence in water and fat in the body. By modifying pulse sequence parameters, it is possible to obtain various contrasts between tissues, which are then converted into diagnostic images. Paramagnetic nanoparticles can function as either T1 or T2 contrast agents by influencing the relaxation properties of water protons. T1 contrast enhancement is most often achieved using gadolinium (Gd^3+^)-based nanoparticles, whereas iron oxide nanoparticles are commonly applied as T2 contrast agents. A major limitation of this strategy is the need to ensure selective accumulation of the contrast agent within the target tumor tissue to obtain accurate and specific imaging results [90].

Manganese-containing silica nanostructures have also attracted considerable interest. Hollow mesoporous carbon nanoparticles incorporating Mn2+ ions [23] enabled pH-responsive MRI enhancement and ultrasound imaging, providing information about the tumor microenvironment. The presence of ionized manganese species facilitates strong interactions with surrounding water molecules, thereby significantly improving the efficiency of T1-weighted MRI contrast enhancement. The study demonstrated the advantages of using inorganic nanoparticles for ultrasound imaging due to their high stability under ultrasonic exposure. They generated enhanced ultrasound signals owing to their excellent echogenic properties, which result from their unique hollow nanostructure. It was shown that HMCNs produced a clear grayscale image enhancement in both harmonic imaging mode and standard B-mode.

Gadolinium is particularly well-suited for theranostics as it acts as both a T1-weighted MRI contrast agent and a radiosensitizing agent. To harness these properties, it was incorporated into an ultrasmall (~5 nm) silica-based nanoparticle (SiBiGdNP), enabling dual MR/CT imaging and radiation enhancement [91].

### 4.4. Multimodal Imaging Platforms

The combination of multiple imaging modalities within a single mesoporous silica nanoparticle system has emerged as an attractive strategy for improving diagnostic accuracy and therapeutic monitoring. Such multifunctional platforms provide complementary information regarding nanoparticle biodistribution, tumor targeting, and anatomical localization. Hybrid PET/CT and SPECT/CT systems have become particularly valuable because they combine highly sensitive molecular imaging with precise anatomical information. Likewise, PET/MRI systems offer simultaneous visualization of biological processes and soft-tissue structures, enabling more comprehensive characterization of nanoparticle behavior. The integration of PET, SPECT, MRI, CT, and optical imaging functionalities into a single MSN system facilitates more accurate assessment of pharmacokinetics, biodistribution, and therapeutic efficacy. Consequently, multimodal imaging is expected to play an increasingly important role in the development of next-generation MSN-based theranostic platforms.

EuDPA/SiO_2_−NH_2_ nanoparticles were converted into a dual-modality agent for SPECT imaging and radiotherapy by partially substituting Eu^3+^ with ^177^Lu^3+^. The ^177^Lu labeling efficiency was quantified, followed by in vivo SPECT/CT imaging and biodistribution studies in mice to evaluate tumor uptake. Therapeutic efficacy was assessed using an HT-29 human colon cancer xenograft model. The results demonstrate that ^177^Lu−EuDPA/SiO_2_−NH_2_ nanoparticles possess both diagnostic and therapeutic potential for targeted colon cancer treatment [92].

Table 4 summarizes representative mesoporous silica nanoparticle (MSN) systems, their imaging modalities and probes, as well as their main purposes and key findings in molecular imaging studies.

### 4.5. Confocal Laser Scanning Microscopy (CLSM)

By screening a selection of mesoporous silica microparticles and then developing appropriate chelation systems, it was possible to synthesize multifunctional mesoporous silica materials such as SBA-15 (Santa Barbara Amorphous type material No. 15) and its spherical analog (sSBA-15) [93]. The resulting particles can be functionalized at the pore surface with the {M(CO)_3_}^+^ complex (where M = ^99m^Tc or Re); of these, the bisquinoline/{M(CO)_3_}^+^ system (M = Re, ^99m^Tc) was found to be suitable for the development of theranostic nanoplatforms intended for (nuclear) medical applications. Therefore, a bisquinoline/{M(CO)_3_}^+^ system (M = ^99m^Tc, Re) was combined with the theranostic capabilities of the ^99m^Tc/^186^/^188^Re pair on a mesoporous SBA-15 silica support; functionalization was optimized using bisquinoline-silanes and surface modification was confirmed by confocal laser scanning microscopy (CLSM). The resulting nanoplatform is suitable for both radiotherapy and chemotherapy, and is being evaluated in in vitro and in vivo studies. CLSM is a fluorescence microscopy technique that generates high-resolution images by scanning a tightly focused laser beam across a sample in a point-by-point, diffraction-limited manner, allowing the visualization of structures labeled with fluorescent probes [94]. Modern confocal microscopes incorporate high-power laser sources, selectable excitation wavelengths, one or more electronic detectors, and a beam scanning system. These components operate in an integrated manner to generate high-resolution digital images. Confocal microscopy, similar to wide-field fluorescence microscopy, is based on fluorescence optics. However, in contrast to wide-field techniques, which illuminate the entire sample simultaneously, confocal microscopy focuses laser light onto a diffraction-limited spot at a defined depth within the sample [95]. In clinical practice, corneal confocal microscopy is extensively applied in the diagnosis of ocular disorders. This advanced, non-invasive in vivo ophthalmic imaging modality allows for rapid and precise evaluation of small nerve fiber damage and regeneration, including in neurodegenerative conditions such as Parkinson’s disease [96]. Table 5 summarizes radiolabeling performance parameters, including radiochemical yield, radiochemical purity, and radiochemical stability, comparing chelator-free and chelator-based approaches.

As expected, nanoparticles exhibit pronounced hepatic uptake, which can be attributed to their recognition and internalization by macrophages present in the liver that are responsible for clearing nanostructures from circulation. Liver macrophages possess a pronounced ability to interact with circulating nanoparticles compared with most target cells and tissues in the body [97].

High radiolabeling efficiency theoretically enables molecules or particles to be employed in in vivo investigations. In addition, the quantitative capabilities of nuclear imaging techniques (e.g., PET standardized uptake values) enable precise dosimetry, supporting individualized treatment planning and minimizing radiation exposure to healthy tissues [98].

Thin-layer chromatography (TLC) is an important method for evaluating the radiochemical yield of labeled compounds, as it is rapid, reliable, and easy to perform. However, the presence of radiochemical impurities remains a drawback in nuclear medicine, often resulting in poor-quality images [99].

## 5. Drugs Loaded into Radiolabeled Mesoporous Silica Nanoparticles

The large specific surface area of mesoporous silica nanoparticles, compared with other types of nanoparticles, enables efficient encapsulation of active substances within a relatively small nanoparticle mass, which results in reduced cytotoxicity. Silica is characterized by excellent biocompatibility; as such, silica-based nanoparticles have been widely applied as drug delivery systems [100]. Silica-based systems serve as versatile platforms for the development of multifunctional nanoplatforms, enabling PET image-guided drug delivery and cancer therapy [101]. Thanks to its potential for non-invasive imaging of neovascularization and guided delivery of antiangiogenic therapeutics; PET has the capability to improve patient prognosis through significantly earlier cancer diagnosis.

One study determined the antibacterial activity of synthesized radionanoparticles and evaluated their potential as drug delivery platforms [27]. Briefly, the antibiotic agent ciprofloxacin was loaded into particles made of mesoporous silica (SBA-16) or silica composed of calcium phosphate (SBA-16/HA). Ciprofloxacin loading into SBA-16APTES and SBA-16/HAAPTES composites was carried out by immersing 100 mg of the powder in a 1000 μg/mL drug solution and maintaining the mixture under magnetic stirring for 48 h. The ciprofloxacin solution was adjusted to the appropriate pH to ensure complete solubility. UV-VIS spectroscopy was used to determine the bound mass of ciprofloxacin within the SBA-16APTES and SBA-16/HAAPTES structures. The amount of drug in the supernatant was measured before and after drug incorporation. Based on the conducted studies, it was demonstrated that the degree of encapsulation of ciprofloxacin is 28.5% for SBA-16APTES and 30.6% for SBA-16/HAAPTES. A likely cause of the similar results was the surface modification of both structures using the APTES reagent. Thermogravimetry analysis (TGA) was also performed. Release studies of ciprofloxacin from SBA-16APTES and SBA-16/HAAPTES showed that 53% of the antibiotic was released from the SBA-16APTES samples within 76 h, and 51% from the SBA-16/HAAPTES samples. The portion of ciprofloxacin present on the surface of the composites was rapidly released, as evidenced by a sharp increase in drug release during the first 9 h.

Curcumin has been reported to inhibit the formation of amyloid fibrils and toxic oligomeric forms. However, its extensive therapeutic potential is constrained by its hydrophobic properties, leading to poor solubility in water and limited bioavailability [102]. To address this limitation and enhance the bioavailability and therapeutic efficacy of curcumin, it was loaded into the pores of MSN-DTPA-Chal. The study involved the synthesis of surface-modified mesoporous silica nanoparticles that could be used in imaging studies and for the treatment of traumatic brain injury [103]. The MSNs were functionalized by anti-inflammatory chalcone (Chal) and DTPA. MSN-DTPA-Chal was radiolabeled with radioisotope ^99m^Tc. MSN-DTPA-Chal was additionally loaded with curcumin. The loading and encapsulation efficiencies were estimated at 88.4 ± 2.6% and 51.3 ± 1.2%, respectively. The amount of adsorbed curcumin was quantified by measuring the absorbance of the supernatant at 420 nm. The dialysis method was used to optimize the in vitro release of curcumin. Cur@MSN-DTPA-Chal (5 mg) was dispersed in 2 mL of PBS (pH 7.4). The mixture was placed in a dialysis tube made of regenerated cellulose (12–14 kDa). The nanoparticles exhibited a two-phase release profile, with 70 ± 2.4% of the encapsulated curcumin released within the first 10 h, followed by a prolonged, sustained release lasting up to 48 h. The initial rapid release is likely due to curcumin molecules situated near the surface or in easily accessible areas of the nanopores. After this burst, the curcumin embedded deeper within the nanopores is released more gradually, resulting in extended, sustained delivery over time. Controlled drug release offers numerous therapeutic benefits, such as optimized drug efficacy and reduced dosing frequency. It improves patient adherence and effectiveness of therapy. Immediate effects can manage acute symptoms, while sustained release provides continuous protection and inhibition of disease progression. As a polyphenol, curcumin has low water solubility and tends to form aggregates. However, when loaded into nanocarriers and exposed to an aqueous environment, it is released slowly. This controlled release ensures a steady availability of curcumin in the water-based medium for different therapeutic uses.

Another study presents the design of a pH-responsive nanocarrier. Its purpose is the targeted delivery of drugs in chemotherapy, which may also find applications in diagnostic imaging [104]. The Fe_3_O_4_@SiO_2_ core–shell nanoparticles were first synthesized. The next step was functionalization with APTES, followed by grafting with the DTPA ligand. Finally the anticancer drug doxorubicin (DOX) was conjugated with Fe_3_O_4_@SiO_2_. The carboxyl groups of DTPA were covalently linked to the amino group in the doxorubicin molecule. The acidic pH (~5.5) led to the breakdown of the amide bond between the doxorubicin molecule and the ligand. As a result, the drug was released gradually over 4 h (85%). The radiolabeling of the carrier was performed by chelating Ga(III) ions with DTPA, which is suitable for use in PET studies. DTPA was used to label the radiotracer by chelating Ga(III) ions. TGA was used to evaluate the quantity of mass loading of organic species on the surface of Fe_3_O_4_ @SiO_2_. The loading of DOX was estimated to be 34 wt%. The drug release behavior of DOX-conjugated Fe_3_O_4_@SiO_2_–APTES–DTPA core–shell nanos was studied at two different pH levels using UV–Vis spectroscopy. DOX release was monitored at a wavelength of 223 nm. The amide bond cleavage is strongly pH-dependent, with acidic conditions (lower pH) promoting a faster release of the drug from the carrier. At pH 5.5, the release of DOX (30.3%) was approximately four times greater than at pH 7.4 (7.6%) during the first 4 h. Lowering the pH of the medium enhanced the amount of DOX released, due to the pH-sensitive cleavage of the amide bonds linking DOX to DTPA.

Agrawal et al. [105] synthesized highly luminescent, biocompatible, and water-dispersible monodispersed NaYF4:Ho,Yb,Li (YHYL) upconversion nanoparticles which were subsequently encapsulated within mesoporous silica (YHYL@m-SiO_2_). DOX was loaded into YHYL@m-SiO_2_ NPs and Folic acid was conjugated to the surface. Then NPs were radiolabeled with 177Lu, a β-emitting radionuclide. Doxorubicin was loaded into the nanoparticles with a 1:10 ratio (wt%) [106]. Doxorubicin is likely to be loaded into the pores of mesoporous silica nanoparticles because of the high porosity of YHYL@m-SiO_2_. In contrast, within YHYL@m-SiO_2_-NH_2_-FA nanoparticles, doxorubicin is expected to undergo relatively weak electrostatic interactions between its protonated −NH_3_^+^ groups and the free carboxyl (COOH) moieties of folic acid, in addition to being physically adsorbed within the mesoporous silica framework. The quantity of DOX encapsulated within the nanoparticles was determined by analyzing the absorption spectra of the supernatant collected after the drug loading process. In vitro drug release kinetics was performed considering two different pH conditions (pH 7.4 and 5) at 37 °C in a reservoir-sink system. At pH 5, the maximum drug release was approximately 90% for YHYL@m-SiO_2_-DOX and about 83% for YHYL@m-SiO_2_-NH_2_-FA-DOX, respectively. At pH 7, drug release is 20% or lower, which is significantly reduced compared to that observed at pH 5. The amine group (NH_2_) of doxorubicin becomes protonated to NH_3_^+^ in the slightly acidic microenvironment of cancer cells (∼pH 5). Consequently, the strength of doxorubicin’s interaction with the nanoparticle surface decreases, which facilitates its release.

Another study reported the development of multifunctional silica nanoparticles (SiNPs) labeled with ^99m^Tc, designed for early in vivo imaging and treatment of HER2-positive breast cancer lesions [107]. A half-chain of trastuzumab (Hc-TZ), a humanized antibody, was conjugated with SiNPs to target HER2 overexpressing tumor cells. SiNPs-TZ were first loaded with fluorescein isothiocyanate (FITC) dye. The histidine residues of the nanocarriers were then radiolabeled with ^99m^Tc and used for ex vivo and in vivo biodistribution analysis. Doxorubicin was additionally incorporated into the nanoparticles to evaluate their in vitro and ex vivo/in vivo delivery performance in comparison with liposomal doxorubicin. The ^99m^Tc-labeled SiNPs were investigated using a HER2-positive tumor xenograft model. Remarkably, doxorubicin was attached to the exterior of the silica core via an isocyanatopropyl trimethoxysilane–drug complex. Drug conjugation was evaluated using UV-Vis spectroscopy. Drug loading efficiency (DLE) and encapsulation efficiency (DEE) were determined to be approximately 1.5–1.6% and 40%, respectively.

İçhedef et al. [26] present a potentially effective radiolabeled drug delivery platform based on methotrexate (MTX), an anticancer compound applied in breast cancer therapy. Mesoporous silica nanoparticles were modified with DTPA, and MTX was attached into the surface. Subsequently, the MTX-MSN nanocomposites were successfully labeled with technetium-99m (^99m^Tc), achieving a radiolabeling efficiency of 92.20 ± 0.8%. Their effects on apoptosis, cytotoxicity and cell uptake were determined against estrogen-positive (ER+) MCF-7 and estrogen-negative (ER−) A549 breast cancer cells. A mixture was prepared by dispersing 25 mg of MSN-DTPA and 5 mg of methotrexate in 5 mL of ethanol, followed by overnight stirring at room temperature. The drug loading capacity of the mesoporous silica nanoparticles was quantified using high-performance liquid chromatography (HPLC). It was found that MTX was loaded to MSN with a high rate of 95%. The high loading capacity of MTX to MSN was favorable for designing an effective drug delivery system.

The anti-angiogenesis drug sunitinib was loaded into hollow mesoporous silica nanoparticles (HMSNs) for tumor vasculature targeted drug delivery and PET imaging, with NOTA used as a macrocyclic chelator [108]. Moreover, the surface of the HMSNs was functionalized with PEG. The cyclo(Arg-Gly-Asp-D-Tyr-Lys) (cRGDyK) peptide was conjugated to the nanoconjugate, which was subsequently radiolabeled with ^64^Cu for PET imaging purposes. Enhanced tumor-targeted delivery of sunitinib was observed. To load HMSN with the hydrophobic drug sunitinib, 0.8 mg of HMSN was dispersed in a 1 mg/mL drug solution in DMSO. UV–visible spectrometry was used to quantify the amount of drug loaded (absorption peak ~440 nm). The drug release behavior was studied in both PBS and DMSO. In DMSO, over 85% of the drug was released from HMSN within just 1 h. This indicates that the drug is unlikely to be released while circulating in the bloodstream or in non-target organs, which helps reduce toxicity and potential side effects during delivery to the tumor site.

Tunçel et al. [44] assessed the effectiveness of a trastuzumab-targeted ^177^Lu-labeled mesoporous Carbon@Silica nanostructure (DOTA@TRA/MC@Si) for HER2-positive breast cancer treatment. The DOTA@TRA/MC@Si nanocomposite was radiolabeled with ^177^Lu with high efficiency, achieving a radiochemical purity of 93.0 ± 2.4%. Subsequently, the in vitro cellular uptake of the radiolabeled nanocomposites was evaluated using SK-BR-3, BT-474, and MDA-MB-231 cell lines. Trastuzumab (TRA) was incorporated into the DOTA@PEI-MC@Si nanocomposite via an amidation reaction [109]. The concentration of TRA attached to the nanocomposite structure was determined using the Bicinchoninic Acid (BCA) assay. The conjugation yield of trastuzumab to the DO-TA@PEI-MC@Si nanocomposite was calculated to be 31.1 ± 4%.

## 6. Regulatory Aspects of Radiopharmaceutical and Nanomedicine Development

Silicon is an established trace element present in the human body, while silica (SiO_2_) has been classified by the FDA as “generally recognized as safe” (GRAS) (ID: 14808-60-7) [110].

Radio-conjugated nanoparticles represent a distinct regulatory category, as they are subject to requirements applicable to both nanomedicines and radiopharmaceuticals. Evidence of quality, safety, and efficacy are required by agencies such as: the FDA, EMA or IAEA (International Atomic Energy Agency).

The parameters used to assess the safety and quality of radiolabeled nanoparticles are summarized in Table 6.

According to FDA guidelines, radiation dose estimates should be derived from animal biodistribution studies using appropriate dosimetric models, while considering recommendations from relevant radiological protection authorities. Furthermore, the radiolabel should remain chemically and metabolically stable to ensure reliable detection and quantification of both the parent compound and its metabolites; if necessary, multiple labeling sites may be employed. Preferably, the total recovery of radioactivity in urine and feces should exceed 90 percent of the administered dose. Analytical methods used in mass balance studies should be selected based on study objectives and properly validated. In general, a combination of radiolabeled and non-radiolabeled techniques is applied: radioactivity is measured in relevant biological matrices using methods such as liquid scintillation counting, accelerator mass spectrometry, or radio-HPLC, while the parent compound and its metabolites are quantified using sensitive, validated LC-MS/MS assays [112].

Regulatory guidelines for nanomedicines commonly define acceptable physicochemical parameters, including particle sizes of 10–200 nm, zeta potential limits around ±30 mV, and polydispersity indices below 0.3. Safety requirements also include low hemolytic activity (<5%), detailed pharmacokinetic characterization, and assessment of organ distribution, with typical accumulation levels of 20–40% in the liver and spleen. Manufacturing consistency is expected to keep batch-to-batch variation within ±10%. The use of Quality by Design (QbD) approaches is recommended, as it may reduce regulatory review times by approximately 15–20% [113]. Fewer than approximately 10% of nanomedicine candidates advance to clinical trials, largely due to inconsistent characterization and insufficient regulatory preparedness. In addition, the absence of standardized terminology, validated testing methods, and certified reference materials further hampers regulatory approval, highlighting the need for global harmonization and dedicated protocols for nanoparticle evaluation [90].

The clinical translation of most reported mesoporous silica nanoparticles (MSNs) has been hindered by suboptimal in vivo pharmacokinetics, characterized by limited tumor accumulation efficiency, pronounced uptake by the reticuloendothelial system (RES), including the liver and spleen, as well as sluggish biodegradation and clearance.

## 7. Conclusions and Further Perspective

Nanotheranostics focuses on the design of nanoparticles for combined therapeutic and imaging applications, enabling their targeted accumulation in diseased tissues, controlled release of the encapsulated active substance, generation of a detectable imaging signal, and subsequent complete elimination from the body without inducing off-target effects or significant toxicity. While enabling targeted therapy, radiolabeled mesoporous silica nanoparticles also allow real-time monitoring of disease progression. It facilitates image-guided therapy, thereby improving the precision of treatment delivery. Targeted drug delivery is particularly important in the case of chemotherapeutic agents, as it increases treatment efficacy, while simultaneously reducing adverse effects [114]. However, the heterogeneous nature of tumors, characterized by diverse cell populations and microenvironments, often impedes uniform nanoparticle distribution and uptake. In addition, biological barriers such as the blood–brain barrier further complicate efficient drug delivery. These factors collectively reduce therapeutic efficacy and highlight the need for improved targeting strategies and more advanced nanoparticle designs to enhance treatment precision [115].

Recent advances in nanomedicine have increasingly aimed to unify diagnostic and therapeutic functions into the discipline of theranostics [116]. It is clearly desirable for a treatment strategy to have theranostic potential [117], i.e., enabling both the diagnosis and treatment of a disease or its progression. For example, biomedical imaging techniques can be combined with therapeutic interventions, particularly in Oncology [118].

The theranostic approach is pivotal to personalized cancer therapy, as it enables patient stratification based on the likelihood of response to specific treatments; it can also facilitate early monitoring of therapeutic outcomes and support prognosis. In nuclear medicine, this strategy involves the use of a single molecular probe, labeled with either diagnostic or therapeutic radionuclides, thereby integrating imaging and treatment within the same platform [119].

Theranostic nanoparticles hold significant promise for advancing cancer management through personalized medicine. However, while numerous theranostic nanoconstructs have achieved encouraging outcomes in preclinical studies, their clinical translation remains limited due to suboptimal targeting and therapeutic efficacy [120].

Nanomaterials can be radiolabeled by the use of BFCs, encapsulation or by chelator-free methods, with the choice of strategy significantly influencing the physicochemical properties of nanoparticles; these can be quantified by measuring hydrodynamic size, zeta potential, and surface characteristics. The stability, systemic circulation time and receptor-specific targeting potential of nanoparticles can be enhanced though surface functionalization with moieties, such as amino groups, monoclonal antibodies, or amino acids [26]. The stability of nanoparticles remains a key concern, as they may undergo degradation, aggregation, or loss of functional properties over time, thereby reducing their therapeutic or diagnostic performance [121].

The selection of radionuclides and chelators is crucial for the integration of radioisotopes with nanomedicine [122] and requires consideration of half-life, bioavailability, compatibility, and biosafety. The half-life determines their application, influencing both diagnostic and therapeutic performance, while an appropriate radionuclide–chelator pairing enhances the efficiency and safety of procedures [123]. Short-lived isotopes are mainly used for imaging, whereas longer-lived ones are preferred for therapy. The choice of radionuclides should be tailored to the specific application. Imaging modalities such as MRI, CT, PET, and fluorescence imaging are improved by nanoparticles containing contrast agent.

Conventional radiotherapy utilizes an external radiation source, which inevitably exposes surrounding healthy tissues to ionizing radiation. In contrast, radiopharmaceutical therapy enables the selective localization of radiation within target cells, thereby substantially minimizing off-target toxicity and adverse effects on normal tissues. Even a small dose of a targeted carrier can provide a safe and effective therapeutic approach. Moreover, radiopharmaceuticals enable the visualization and identification of drug accumulation within target tissues, which significantly enhances the accuracy and precision of personalized treatment.

The design of nanotheranostic systems can be realized through several distinct strategies. The first approach relies on nanomaterials that intrinsically combine both imaging and therapeutic functionalities within a single nanocarrier. An alternative strategy involves the covalent or non-covalent attachment of imaging or therapeutic agents to nanoparticles that function as delivery platforms. A third approach is based on the encapsulation of drugs within nanocarriers, where subsequent radiolabeling enables non-invasive tracking of their biodistribution, administration pathway, and accumulation within target tissues. However, while positive results have been observed in many preclinical studies, their translation into clinical practice remains limited due to poor targeting precision and insufficient therapeutic efficacy [71]. Research in nanotheranostics, based on mesoporous silica nanoparticles, should be directed toward the rational design of structures, capable of integrating both imaging and therapeutic functions, with particular emphasis on targeted drug delivery strategies. The realization of such systems requires close interdisciplinary collaboration among the fields of pharmacy, medicine, materials engineering, and nanotechnology. Among the key challenges in the development of nanotheranostic agents are their costly and technically complex synthesis and design processes. Scalability remains a major issue, as transitioning from small-scale laboratory synthesis to large-scale production is often difficult due to variations in yield and product quality.

While nanomaterials small enough to undergo renal clearance can mitigate issues associated with long-term toxicity, significant obstacles remain in designing ultrasmall multifunctional nanoparticles that combine adjustable pharmacokinetics with efficient and persistent tumor targeting. Consequently, nanosystems capable of maintaining adequate circulation in the bloodstream, selectively accumulating within tumors, and subsequently disassembling into easily cleared fragments are likely to offer considerable potential for clinical translation.

In 2014, Bradbury and co-workers reported the first-in-human clinical evaluation of 124I-labeled ultrasmall SiNPs (Cornell dots, C dots) in patients with metastatic melanoma. This milestone study provided compelling evidence supporting the clinical translational potential of radiolabeled SiNPs and has substantially accelerated research efforts in this field [124].

## Figures and Tables

**Table 1:** Radioisotopes employed in silica nanoparticle radiolabeling: physical properties and applications.

Radioisotope	Half-Life (T ½)	Emission Type	Application	Ref.
^99m^Tc	6.02 h	γ (gamma emission)	SPECT imaging	[23,24,25,26,27,28,29]
^45^Ti	3.08 h	β^+^ (positron emission)	PET imaging	[30]
^11^C	20.4 min	β+	PET imaging	[31]
^89^Zr	78.4 h	β^+^	PET imaging	[32,33,34,35,36,37]
^131^I	8.01 days	β^−^ (negatron emission) + γ	PET-CT/SPECT imaging, radioiodine therapy	[36,37,38]
*As(III) (* = 71, 72, 76, 74)	^71^As 26.0 h ^72^As 426.5 h ^73^As 80.3 d ^74^As 17.7 d	^71^As: β^+^ ^72^As: β^+^ ^73^As: EC (electron capture) ^74^As: β^+^, β^−^	PET imaging	[38]
^18^F	109.8 min	β+	PET imaging	[28,29,30,31,32,33,34,35,36,37,38,39,40]
^64^Cu	12.7 h	β+ + β−	PET imaging and theranostic	[41]
^68^Ga	1.1 h	β^+^	PET imaging, PET with a dual-head SPECT system	[42,43]
^177^Lu	6.7 d	β^−^ + γ	Targeted radionuclide therapy	[44]
^56^Co	77.26 d	β+/EC	PET imaging	[45,46]

*As(III) represents arsenic isotopes with mass numbers 71, 72, 74, and 76.

**Table 2:** Surface-modified mesoporous silica nanoparticles, labeled with various isotopes, for advanced applications.

Radionuclide	Radiolabeling Mediator	Type of Silica Nanoparticle	Surface Modification (Linkers)	Application	Ref.
^99m^Tc	p-SCN-Bn-DTPA	Manganese oxide-based mesoporous silica nanoparticles	mPEG 5000 –NHS fluorescein isothiocyanate—FITC (for the in vitro experiments)	Nanotheranostics combining dual-modal imaging with anticancer therapeutic properties.	[23]
^99m^Tc	Chelator-free	Mesoporous silica (with magnetic core)	Monoclonal antibodies (Mab)—trastuzumab	Verification of indirect entrapment efficiency using radiolabeling.	[24]
^99m^Tc	DTPA	SBA-16 mesoporous silica nanoparticles	-	Determining the fate of the particles in healthy mice.	[23]
^99m^Tc	DTPA	Uniform mesoporous silica nanoparticles	3-aminopropyltriethoxysilane (APTES)	Developing a biocompatible ^99m^Tc- radiolabeled drug delivery nanosystem for methotrexate allowing tumor imaging.	[28]
^99m^Tc	DTPA	Mesoporous silica SBA-16/hydroxyapatite-based composite	APTES	Evaluating the biological activity of silica/hydroxyapatite composite (ciprofloxacin loaded), against susceptible bacteria. Evaluation of these systems as a matrix for controlled drug delivery.	[27]
^99m^Tc	Chelator-free	Magnetic core mesoporous silica nanoparticles	Trastuzumab monoclonal antibody	Intralesional nanodrug for breast cancer imaging and possible therapy.	[28]
^99m^Tc	Chelator-free	Magnetic core mesoporous silica nanoparticles	Lack of surface modifications	Determination of potential toxicogenic risk on embryonic/fetuses in animal model.	[27]
^45^Ti	Chelator-free	Mesoporous silica nanoparticles	Amine groups attaching by APTES PEG5k-succinimidyl carboxymethyl ester (PEG5k-SCM)	In vivo tumor-targeted PET imaging in 4T1 murine breast tumor model.	[30]
^11^C	Radiolabeling methanol	Iron-oxide modified mesoporous silica MCM-41	Iron nitrate or acetylacetonate precursors	Elucidation the influence of various methanol conversion reaction pathways on the state of the iron species in modified MCM-41 materials.	[31]
^89^Zr	Chelator-free	Dendritic mesoporous silica nanoparticles (bMSNs), with a centrally radiating, two-tiered porous network	Amine groups attaching by APTES PEG5k-succinimidyl carboxymethyl ester (PEG5k-SCM) anti-CD105 antibody, (TRC105)	Carrying small-molecule and large biomolecular drugs for combination therapy. Targeting multiple tumor types. Self-destruction to allow excretion from the body.	[32]
^89^Zr	p-SCN-deferoxamine (DFO)	Pore expanded mesoporous silica spheres	Amine groups attaching by APTES Alexa Fluor 488—bright, green-fluorescent dye Poly(ethylene glycol)-succinimidyl succinate	Evaluation of the biodistribution of ^89^Zr-labeled mesoporous silica nanoparticles (MSNs) using a prostate cancer mouse model bearing LNCaP C4-2 and PC-3 tumor xenografts with focus on passive targeting.	[33]
^89^Zr	Chelator-free	Mesoporous silica nanoparticles (MSNs) with controllable mesoporous structure	Lack of surface modifications	Studying the in vivo long-term fate of MSN and PET image-guided drug delivery.	[34]
^89^Zr	Chelator-free	Hollow mesoporous silica nanoparticles	Amine groups attaching by APTES	Using radionuclides to activate a well-known photosensitizer (chlorin e6, Ce6) for therapeutic purposes.	[35]
^89^Zr, ^131^I	Chelator-free	Aminated mesoporous silica nanoparticles (MSNs)	Homing peptide, Lin-TT1 (AKRGARSTA) NHS-PEG 5000-maleimide. Tyrosine (131I)	Demonstrating the biodistribution of the radiolabeled MSN. Demonstrating the necessity to choose an appropriate pathway for labeling a nanocarrier.	[36]
^131^I	Iodination tube	Zn phthalocyanine-loaded mesoporous silica (MSNP5)	Thiol functional groups (3-mercaptopropyl)trimethoxysilane PEGylated with Mal-PEG-COOH Cetuximab antibody	Synthesizing a multifunctional agent that can be used effectively in photodynamic therapy of pancreatic cancer, and to enable imaging with radiolabeling.	[35]
^131^I	Chelator-free	Mesoporous silica-based urease-powered nanobots	Amine groups attaching by APTES Glutaraldehyde Urease PEG Gold nanoparticles (AuNPs)	Potential use of urease-powered nanobots as efficient therapeutic carriers for bladder cancer therapy.	[38]
^18^F	Fluoronicotinic acid-2,3,5,6-tetrafluorophenyl ester	Mesoporous silica-based urease-powered nanobots	Amine groups attaching by APTES Glutaraldehyde Urease PEG Gold nanoparticles (AuNPs)	Testing radiolabeled mesoporous silica-based urease-powered nanobots in an orthotopic mouse model of bladder cancer.	[28]
^18^F	N-Succinimidyl 4-fluorobenzoate (SFB)	Mesoporous silica nanoparticles	Amine groups attaching by APTES	Evaluating in vivo biodistribution of ^18^FMSiNPs by PET.	[39]
*As(III) (* = 72, 76, 74, 71)	Chelator-free	Thiolated mesoporous silica nanoparticle	Thiol or amine functional groups through reaction with (3-mercaptopropyl)-trimethoxysilane and (3-aminopropyl)trimethoxysilane respectively	Preparation of theranostic radiopharmaceuticals and image-guided drug delivery.	[38]
^64^Cu	p-SCN-Bn-NOTA	Biocompatible mesoporous silica nanoparticles	Thiol groups (-SH) Mal-PEG 5k-NH Mal-PEG 5k-SCM TRC105 antibody (specific for CD105/endoglin)	Accumulation prominently at the 4T1 tumor site. Targeted delivery of doxorubicin (DOX) in *4T1* tumor-bearing mice. Potential for future image-guided drug delivery and targeted cancer therapy.	[41]
^68^Ga	Chelator- free	Pyridine functionalized mesoporous silica MCM-41	3-chloropropyltrimethoxysilane 4-methylpyridine	Production of drug delivery systems for theranostic applications.	[42]
^68^Ga	Chelator-free	MCM-41 mesoporous silica	fluorescent cadmium telluride quantum dotsthiol (–SH) groups	PET/fluorescence dual-modality imaging.	[43]
^177^Lu	DOTA-NHS ester	Mesoporous Carbon@Silica nanostructure	Trastuzumab	Treatment of HER2-positive breast cancer.	[44]
^56^Co	Chelator-free	Amorphous silica NPs	No surface modifications	Developing a method for synthesizing silica NPs with ^56^Co as an incorporated extrinsic radiotracer that can be easily separated from the target material. Its half-life (77.26 days) and intense gamma emission make it suitable for tracing experiments.	[45]

*As(III) represents arsenic isotopes with mass numbers 71, 72, 74, and 76.

**Table 4:** Representative molecular imaging studies employing radiolabeled mesoporous silica nanoparticles.

Representative MSN System	Imaging Modality	Imaging Probe	Main Purpose	Major Findings
PEGylated ^45^Ti-MSN [25]	PET	^45^Ti	Biodistribution and pharmacokinetics	Predominant liver accumulation with measurable tumor uptake.
^89^Zr-dMSN-TRC105 [32]	PET	^89^Zr	Long-term in vivo tracking	High radiostability and prolonged circulation.
PEGylated ^89^Zr-MSN [33]	PET	^89^Zr	Tumor targeting evaluation	Higher uptake in LNCaP C4-2 than PC-3 tumors.
^18^F-MSiNPs [39]	PET	^18^F	Biodistribution studies	Accumulation in RES organs and lung capillaries.
^64^Cu-NOTA-mSiO_2_-PEG-TRC105 [41]	PET	^64^Cu	PET-guided drug delivery	High tumor uptake and hepatobiliary clearance.
^99m^Tc-MnOx-MSN-PEG [23]	SPECT/MRI	^99m^Tc + MnOx	Dual-modality imaging	Enhanced tumor visualization with high radiochemical stability.
^99m^Tc-MSN-APTES-DTPA [24]	SPECT	^99m^Tc	Biodistribution analysis	High uptake in liver and lungs.
^99m^Tc-MTX-MSN [24]	SPECT	^99m^Tc	Drug delivery monitoring	High labeling efficiency and prolonged stability.
^177^Lu-EuDPA/SiO_2_-NH_2_ [44]	SPECT/CT	^177^Lu	Theranostics	Effective tumor visualization and therapeutic activity.
HMCNs [23]	MRI/US	Mn^2+^	Tumor microenvironment imaging	pH-responsive MRI enhancement and ultrasound contrast.
SiBiGdNP [91]	MRI/CT	Gd + Bi	Dual imaging and radiosensitization	Improved tumor control and survival.
MCM-41-NH_2_-DTPA-Gd-MIH [43]	MRI	Gd^3+^	Breast cancer theranostics	Simultaneous imaging and chemotherapy.

**Table 5:** Comparative analysis of chelator-based and chelator-free radiolabeling approaches for silica nanoparticles.

Parameter	Chelator-Free	Chelator-Based	Ref.
Radiochemical yield [%]	73–95	14–99	[23,24,25,26,27,28,29,30,31,32,33,34,35,36,37,38,39,40,41,42,43,44]
Radiochemical purity [%]	95–99	90–98	
Radiochemical stability [%]	85–99	75–98	
Biodistribution pattern [% DI/g]	Nanoparticle-dependent. High uptake in liver, spleen, kidneys, lungs. Typically lower tumor biodistribution.	Nanoparticle-dependent. High uptake in liver, spleen, kidneys, lungs. Ty pically lower tumor biodistribution.	
Ease of radiolabeling	Direct chelator-free radiolabeling approach.	Chelator-conjugated radiolabeling approach.	
Imaging modality	PET, PET-CT, SPECT, MRI, PET with a dual-head SPECT system.	PET, SPECT, SPECT-MRI. Gamma camera imaging.	

**Table 6:** Key characterization, quality control, sterility, and biological evaluation parameters for nanoparticle-based products according to FDA recommendations [111].

Physicochemical Characterization of Nanoparticles	Radiochemical Parameters	Sterility and Biosafety	Biological Evaluation
Chemical composition Average particle size Particle size distribution General shape and morphology Surface properties (e.g., surface area, surface charge, chemical reactivity, ligands, hydrophobicity, and roughness) Physical stability (e.g., aggregation and agglomeration or separation).	Radionuclide identification Radiochemical purity Stability	Endotoxin content Product stability during storage Sterility testing	In vivo biodistribution Dosimetric analysis Immunogenicity Systemic and organ-specific toxicity Pyrogenicity

## Data Availability

No data was used for the research described in the article.

## Abbreviations

AEs	Auger electrons
APTES	3-aminopropyltriethoxysilane
ATO	Arsenic trioxide
AuNPs	Gold nanoparticles
BCA	Bicinchoninic Acid
BFC	Bifunctional chelating agents
Chal	Chalcone
CT	Computed tomography
DFO	Deferoxamine
DLE	Drug loading efficiency
DMSO	Dimethyl sulfoxide
DOX	Doxorubicin
DTPA	Diethylenetriaminepentaacetic acid
ER(-)	Estrogen negative
FDA	Food and Drug Administration
FITC	Fluorescein isothiocyanate dye
Mab	Monoclonal antibodies
MRI	Magnetic resonance imaging
MSNs	Mesoporous silica nanoparticles
MTX	Methotrexate
NODAGA	2-(4,7-bis-(carboxymethyl)-1,4,7-triazonan-1-yl)pentanedioic acid
NODASA	1,4,7-triazacyclononane-1-succinic acid-4,7-diacetic acid
NOTA	1,4,7-triazacyclononane-1,4,7-triacetic acid
NPs	Nanoparticles
PEG	Polyethylene glycol
PET	Positron emission tomography
p-SCN-Bn-NOTA	S-2-(4-isothiocyanatobenzyl)-1,4,7-triazacyclononane-1,4,7-triaceticacid
SBA-15	Santa Barbara Amorphous type material No. 15
SFB	N-Succinimidyl-4-fluorobenzoate
SiNPs	Silica nanoparticles
SPECT	Single photon emission computed tomography
TLC	Thin-layer chromatography
TRA	Trastuzumab
